# Psychosocial Challenges in Mental Health Care for Mothers Bereaved by Suicide

**DOI:** 10.1192/j.eurpsy.2026.11978

**Published:** 2026-07-31

**Authors:** R. Zavrou, A. Charalambous, E. Papastavrou, A. Koutroubas, M. Karanikola

**Affiliations:** Cyprus University of Technoology, Limassol, Cyprus

## Abstract

**Introduction:**

Evidence suggests that suicide-exposed family members are at greater risk of developing mental and physical health problems, including higher rates of psychiatric hospitalization, compared to the general population. Understanding how the suicide of a child or young person shapes parents’ needs and attitudes is crucial for informing interventions that foster effective help-seeking, deliver evidence-based, person-centered care, and guide the adaptation of mental health services. Although a body of research already exists, further qualitative studies are needed to capture the lived experiences of bereaved mothers within specific sociocultural contexts, particularly in Southern Europe and the Eastern Mediterranean region.

**Objectives:**

Given the persistent stigma surrounding suicide in Southern European societies, we explored Greek-speaking suicide-bereaved mothers’ needs in the Republic of Cyprus, aiming to provide data to update understanding of their experiences and the focus of mental healthcare services.

**Methods:**

Inductive content analysis of qualitative data collected through personal interviews with ten mothers were employed. The current study is a secondary analysis of these data, conducted to answer a new research question and offer additional insights that go beyond the boundaries of the original paper.

**Results:**

Participants’ needs centered on a “persistent effort for protection,” encompassing three interconnected domains: (1) protecting themselves from stigma, social judgment, and emotional disintegration; (2) safeguarding the psychological well-being and cohesion of surviving family members; and (3) preserving the posthumous dignity and memory of their deceased child. Rather than seeking formal support, participants overwhelmingly turned away from mental health services, citing a lack of empathy, cultural misunderstanding, and fear of being further stigmatized. Their coping strategies, including self-isolation, emotional suppression, peer-based connection, and legacy-building, were developed independently of clinical support. Mental health professionals were often perceived as inadequate or even harmful, reinforcing the participants’ drive toward self-reliance and autonomy in bereavement. These responses reflect how gendered social expectations and stigma surrounding suicide shape bereaved mothers’ disengagement from healthcare systems, despite their intense psychological needs.

**Conclusions:**

These findings underscore a significant psychosocial barrier to mental health care for women navigating traumatic grief, particularly in sociocultural contexts where suicide remains highly stigmatized.

**Disclosure of Interest:**

None Declared

